# P-2134. Development and Validation of a Quantitative PCR Assay for the Detection of *Mucorales* in Human Serum Specimens

**DOI:** 10.1093/ofid/ofae631.2289

**Published:** 2025-01-29

**Authors:** Jerod Davidson, James Grantham, Clayton Thomas, Jamie Nutt, Pam Morris, Zachary Kockler, Steve Kleiboeker

**Affiliations:** Eurofins Viracor, Lenexa, Kansas; Eurofins Viracor, Lenexa, Kansas; Eurofins Viracor, Lenexa, Kansas; Eurofins Viracor, Lenexa, Kansas; Eurofins Viracor, Lenexa, Kansas; Eurofins Viracor, Lenexa, Kansas; Eurofins Viracor, Lenexa, Kansas

## Abstract

**Background:**

Mucormycosis are diseases with high morbidity and mortality caused by fungal species belonging to the order *Mucorales*. The intended use of a *Mucorales* qPCR assay is as an adjunct to microbiological, clinical, and radiologic means of diagnosing invasive mucormycosis (IM). Serum collection is a non-invasive alternative to BAL and biopsy. Our design process included sequences from all species within the *Mucorales* genera: *Cunninghamella*, *Lichtheimia*, *Absidia*, *Apophysomyces*, *Mucor*, *Rhizomucor*, *Rhizopus* and *Saksenaea*. The *Mucorales* qPCR assay is designed to detect and quantify *Mucorales* DNA extracted from human serum.

**Figure:**
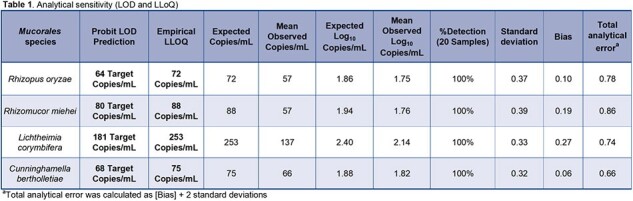

**Methods:**

Primers and probes were designed to target the 18S regions of *Mucorales* species. Nucleic acid was extracted using the MagMAX™ Cell-Free DNA Isolation Kit and KingFisher™ Flex system. Amplification and detection were performed using TaqMan™ Fast Universal Master Mix and the Applied Biosystems™ QuantStudio 7 Pro. Quantification was performed using *Rhizopus oryzae* target-bearing linearized plasmid standards with results reported in copies/mL. Analytical validation was performed by spiking negative human serum with four different *Mucorales* species’ DNA: *Rhizopus oryzae*, *Rhizomucor miehei*, *Lichtheimia corymbifera*, and *Cunninghamella bertholletiae*.

**Figure:**
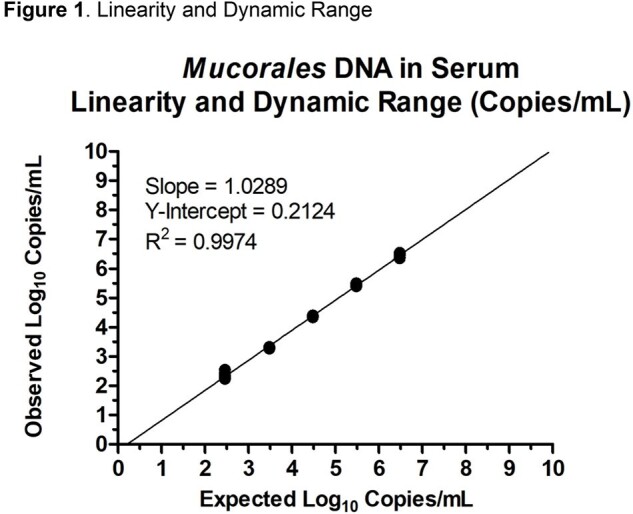

**Results:**

The limit of detection was 181 copies/mL, and the lower limit of quantification was determined to be 253 copies/mL as shown in Table 1. Linear regression of dilution data produced an R^2^=0.997, slope=1.029, and y-intercept=0.212 as show in Figure 1. The *Mucorales* target detection was 100% (60 of 60) of blinded positive samples with all observed log_10_ copies/mL results within ±0.5 log_10_ copies of expected values as shown in Table 2. All (20 of 20) blinded negative samples were negative for *Mucorales* and positive for Internal Control (IC). Intra-assay precision ranged from 5 – 22 %CV and inter-assay precision ranged from 12 – 17 %CV as Shown in Table 3.

**Figure:**
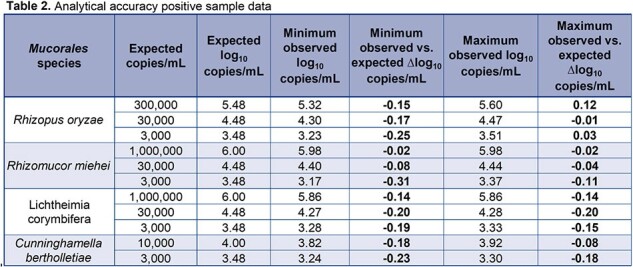

**Conclusion:**

The validated assay described here demonstrates excellent sensitivity, linearity, precision, and accuracy, providing a reliable means of detecting *Mucorales* DNA in human serum. This assay represents a non-invasive, rapid, and sensitive method to guide clinical decisions on patient populations at high risk of IM.

**Figure:**
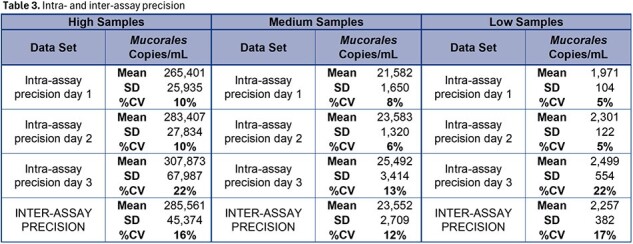

**Disclosures:**

Jerod Davidson, B.A., Eurofins Viracor: Employee James Grantham, B.S., Eurofins Viracor: Employee Clayton Thomas, B.S., Eurofins Viracor: Employee Jamie Nutt, n/a, Eurofins Viracor: Employee Pam Morris, M.S., PMP, Eurofins Viracor: Employee Zachary Kockler, PhD, Eurofins Viracor: Employee Steve Kleiboeker, PhD, Eurofins: employee|Eurofins: Stocks/Bonds (Public Company)

